# Autophagy in Cancer Therapy—Molecular Mechanisms and Current Clinical Advances

**DOI:** 10.3390/cancers13215575

**Published:** 2021-11-08

**Authors:** Ingo Ganzleben, Markus F. Neurath, Christoph Becker

**Affiliations:** 1 Department of Medicine 1, University Hospital, Friedrich-Alexander-Universität Erlangen-Nürnberg, 91054 Erlangen, Germany; ingo.ganzleben@uk-erlangen.de (I.G.); markus.neurath@uk-erlangen.de (M.F.N.); 2 Deutsches Zentrum Immuntherapie (DZI), Universitätsklinikum Erlangen, 91054 Erlangen, Germany

**Keywords:** autophagy, mitophagy, glutamine, cancer, reverse Warburg effect, hydroxychloroquine

## Abstract

**Simple Summary:**

Autophagy is the capability of cells to dismantle and recycle parts of themselves. This process is closely intertwined with other crucial cell functions, such as growth and control of metabolism. Autophagy is oftentimes dysregulated in cancer and offers established and advanced tumors protection against a lack of nutrients and an advantage regarding proliferation. This review will present an overview of the basics of human autophagy, its dysregulation in cancer, and approaches to target autophagy in cancer treatment in recent and current clinical trials as well as new findings of preclinical research.

**Abstract:**

Autophagy is a crucial general survival tactic of mammalian cells. It describes the capability of cells to disassemble and partially recycle cellular components (e.g., mitochondria) in case they are damaged and pose a risk to cell survival or simply if their resources are urgently needed elsewhere at the time. Autophagy-associated pathomechanisms have been increasingly recognized as important disease mechanisms in non-malignant (neurodegeneration, diffuse parenchymal lung disease) and malignant conditions alike. However, the overall consequences of autophagy for the organism depend particularly on the greater context in which autophagy occurs, such as the cell type or whether the cell is proliferating. In cancer, autophagy sustains cancer cell survival under challenging, i.e., resource-depleted, conditions. However, this leads to situations in which cancer cells are completely dependent on autophagy. Accordingly, autophagy represents a promising yet complex target in cancer treatment with therapeutically induced increase and decrease of autophagic flux as important therapeutic principles.

## 1. Autophagy in Cancer Treatment—A Molecular Introduction

Autophagy is a fundamental biological principle describing, in general, the capability of cells to degrade and partially recycle cellular components such as organelles (e.g., mitochondria) or molecules (e.g., proteins). The term was coined in 1963 by the later Nobel laureate Christian de Duve, a pioneer in autophagy research, who among other contributions first described the lysosome [1], a central organelle in the process of autophagy. Autophagy-focused research made another quantum leap when Yoshinori Ohsumi and colleagues characterized the process of autophagy in yeast [2]. In their work, the authors characterized both important regulators of the autophagy cascade, such as target of rapamycin (TOR) [3], as well as the molecular details of autophagy execution [4,5,6]. While autophagy was triggered in these experiments by utilizing a nutrient-deficient environment to induce autophagic degradation of cytosolic components, groundbreaking findings by Klionsky and colleagues led to the understanding that autophagy also had highly specific and targeted aspects, such as the cytoplasm to vacuole targeting (Cvt) pathway that has been elucidated in yeast but does not exist in mammals [7,8,9]. A harmonization of the existing different nomenclatures for players involved in autophagy was drafted in 2003 and led to the currently most commonly used terminology designating autophagy-related genes and proteins (ATG) [10].

Autophagy is oftentimes described as “recycling” performed by the cell when it is confronted with nutrient depletion and other challenging circumstances. Under these conditions, the process of macroautophagy means the unspecific non-selective engulfment of cytosolic components and organelles and their degradation for the purpose of energy production and generation of basic components, which are then both used to sustain critical cell activities, as reviewed previously by Mizushima and Komatsu [11]. Mammalian target of rapamycin (mTOR) together with other cofactors acts as the mTOR complex 1 (mTORC1) in order to sense metabolic conditions, such as nutrient depletion [12], but also endoplasmic reticulum (ER) stress, hypoxia, and other triggers. mTORC1 normally acts as an inhibitor of autophagy by interacting with the complex of Unc-51-like autophagy activating kinase (ULK1)/Atg13/FIP200, leading to the phosphorylation of ULK1 and Atg13. Nutrient depletion-based disinhibition of the ULK1 complex in turn leads to initiation of the macroautophagy cascade [13]. Importantly, AMP-activated protein kinase (AMPK) contributes to this regulation under low-glucose conditions by phosphorylating intermediate proteins such as Raptor to reduce the activity of mTORC1. This, in turn, leads to a reduced phosphorylation of the mTORC1 target ULK1 on Ser 757, which then allows for direct activation of the ULK1 complex by AMPK via phosphorylation on Ser 317 and Ser 377 [14].

As elaborately and conceptually reviewed by Dikic and Elazar [15], phagophore nucleation occurs at the start of the autophagy cascade by phosphorylation of the phosphatidylinositol 3-kinase class III (PI3KC3) complex I, which among others contains beclin 1, Vps34, and Atg14. This complex leads to the accumulation of phosphatidylinositol 3-phosphate (PI3P), which is chiefly responsible for developing the isolation membrane, a structure developing from the ER into a structure that has been named the omegasome. Vps34 and beclin 1, both components of the PI3KC3 complex I, play a major role in this formation process in particular [16]. As Dikic and Elazar further illustrate [15], the WD repeat domain phosphoinositide-interacting proteins (WIPIs) in combination with Zinc finger FYVE domain-containing protein 1 (DFCP1) then interact with PI3P to attract the ATG12~ATG5–ATG16L1 complex, which facilitates the addition of the hallmark protein LC3 (microtubule-associated proteins light chain 3) to the expanding omegasome. LC3-I is then converted to LC3-II, which is a fulcrum for expanding and closing the phagophore membrane. The developing entity, enclosed by a double-layer membrane, is called the autophagosome which, in a last step, fuses with the lysosome to form the autolysosome. Importantly, ATGs are mostly preserved during this process and can be reused [15].

The macroautophagy of mitochondria, a form of mitophagy, is an important and prominent example of targeted autophagy in mammalian cells and hence in humans. Mitophagy by means of microautophagy, a less well-studied form of autophagy which is based on pinocytosis and has been recently reviewed in greater detail by Schuck [17], has been described as an important autophagic mechanism of mitochondria in yeast cells [18]. However, this avenue does not seem to be the predominant mitophagic pathway in mammalian cells, at least as far as is known today. Mitophagy in mammalian cells also hinges on the previously mentioned autophagy fulcrum of LC3. As comprehensively reviewed by Yoo and Jung [19], the molecular interactions of mitochondrial proteins and LC3 can occur via different adaptor proteins. Mitophagy can occur via a well-characterized pathway, including PTEN-induced kinase 1 (PINK1) and Parkin. This pathway is, for example, triggered by mitochondrial membrane depolarization and regulated by mitochondrial proteins such as Phosphoglycerate Mutase Family Member 5 (PGAM5) by incompletely understood mechanisms [20,21,22]. The key features of this pathway include decreased degradation of PINK1 by Presenilin-associated rhomboid-like protein (PARL), PINK1’s translocation to the outer mitochondrial membrane, and subsequent PINK1-mediated recruitment of Parkin, a ubiquitin-ligase, to the damaged mitochondrion [23,24]. Targeted macroautophagy is finally facilitated by Parkin-mediated polyubiquitylation, which connects the mitochondria via various adaptors to the autophagosome membrane. These adaptor proteins include, among others, sequestosome-1 (p62) and optineurin (OPTN), which all share a common LC3-interacting region (LIR) to connect to LC3 besides other autophagy players, and hence to the autophagosome membrane [25,26,27]. A variety of proteins are, however, able to directly function as links between the mitochondrion that they are a part of and the LC3 of the autophagosome membrane, such as Nip3-like protein X (NIX) [28], BCL2/Adenovirus E1B 19 kDa Interacting Protein3 (BNIP3) [29], and FUN14 Domain Containing 1 (FUNDC1) [30], which are of importance in hypoxia-induced mitophagy, as also reviewed by Yoo and Jung [19] and Vara-Perez and colleagues [31]. Importantly, PGAM5 plays a role in FUNDC1-mediated mitophagy by altering the phosphorylation status of FUNDC1 under hypoxic conditions [32].

One of the least understood modes of regulating autophagy has been termed chaperone-mediated autophagy (CMA). CMA, as reviewed recently by Kaushik and Cuervo [33], is in essence the targeted degradation of cytosolic proteins that are recognized by chaperones via a contained KFERQ-like motif. The chaperones, among them heat-shock proteins as hsp70 [34,35], then guide the proteins towards the lysosome where the cargo interacts with Lysosome-Associated Membrane Protein 2 (LAMP2) to initiate degradation [36]. 

**Figure 1:**
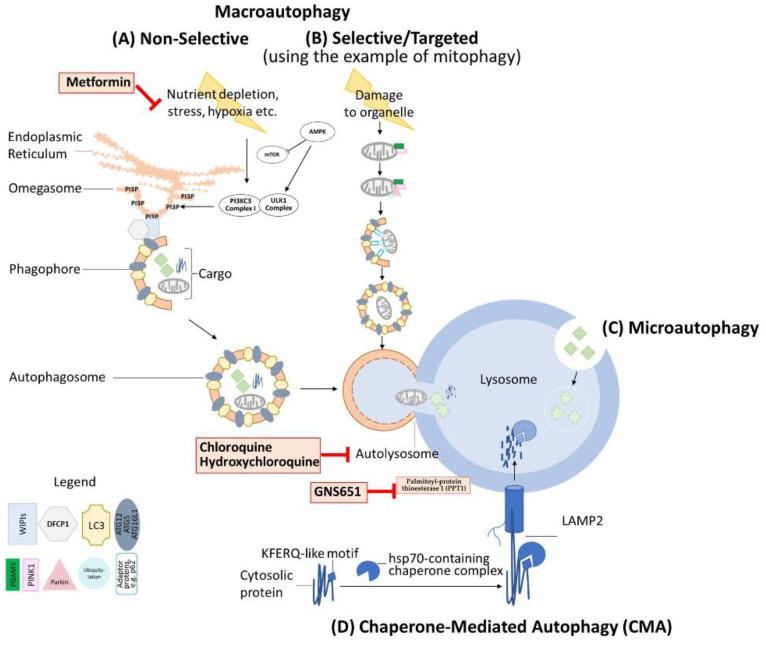
Illustration of micro/macro/chaperone-mediated autophagy. (A) Non-selective macroautophagy (unspecific engulfment of cytosolic components and organelles and their degradation for the purpose of energy production and generation of basic components). (B) Selective/targeted macroautophagy using the example of mitophagy. (C) Microautophagy. (D) Chaperone-mediated autophagy. Autophagy-modulating drugs that have been applied in clinical trials for the therapy of distinct cancer entities, as reviewed in this current manuscript, are indicated at their site of action in the process of autophagy.

A graphical illustration of these mentioned autophagy pathways is displayed in Figure 1. 

Assessing autophagy is a highly complex endeavor because it not only includes multifaceted pathways with myriads of players, but also because it is in a constant state of flux due to its task of degrading cellular components.. To standardize methodical approaches in the study of autophagy over different disciplines, detailed and comprehensive guidelines have been published. One key element of these guidelines emphasizes the importance of accounting for the element of autophagic flux by using autophagy inhibitors [37]. While these fundamentals are important to understand, they are not, at least in detail, the main focus of this review.

## 2. Autophagy in Cancer Treatment—A Double-Edged Sword 

As a fundamental and highly conserved principle throughout species, autophagy is involved in a multitude of physiological functions as well as pathophysiological conditions in humans, as reviewed by Ichimiya and colleagues, including liver, heart, neurological, and pulmonary diseases, as well as cancer [38]. 

Based on the known functions and molecular pathways of autophagy, both tumor suppressive and oncogenic functions of autophagy appear equally plausible. However, the reality seems nuanced and complicated, first and foremost because autophagy is a highly context-dependent process. It depends both on the spatiotemporal context as well as the general (e.g., metabolic) circumstances of a cell whether autophagy in the end overall benefits or harms the cell. The fact that autophagy is a way of cells to function and cope with cellular stressors proves as the often-cited double-edged sword and a mixed blessing for cancer cells. An overview of selected tumor-suppressive and oncogenic functions of autophagy in cancer and the corresponding regulators can be found in Table 1.

### 2.1. Tumor-Suppressive Functions of Autophagy

Hallmark studies by Beth Levine and colleagues revealed the tumor-suppressive function of beclin 1 in murine tumor models with evidence of its role in human disease, such as breast cancer, as well [39,53]. Beclin 1 was not only described to be reduced significantly in a large percentage of breast cancer cell lines and tissues samples, but Levine and colleagues also provided functional evidence that beclin 1 expression resulted in increased autophagy and reduced tumorigenesis in vitro and in a murine tumor model in vivo [53]. Complementary data using a heterozygous beclin 1 knock-out mouse (beclin 1^+/−^) revealed increased tumorigenesis, establishing beclin 1 as a “haplo-insufficient tumor-suppressor gene” [39]. As previously reviewed by Avalos and colleagues [54], other ATGs have been implicated as tumor-suppressor genes, such as Atg7, whose organ specific knock-out led to neoplastic lesions of the liver [40]. This study found that a lack of Atg7 in hepatocytes resulted in several interconnected outcomes, namely autophagy deficiency associated with swelling of the mitochondria, buildup of p62, and subsequently signs of oxidative stress. However, the resulting neoplastic lesions were benign and not malignant [40]. Along the same lines are functional data that Strohecker and White provide based on studies in Atg7-deficient mice undergoing the BrafV600E-induced lung cancer model [41]. They propose a model of autophagy-mediated tumor suppression by detoxification of oxidative stress and damage, as Atg7 deletion led to increased levels of oxidative stress and promoted tumor formation and proliferation at least initially. In contrast, Atg7 deletion in this model in the long term resulted in reduced tumor burden and a decreased proliferative capacity. The authors speculate whether the accumulation of defective mitochondria due to reduced mitophagic degradation might be a reason for their observation. Importantly, however, addition of external glutamine partially reconstituted tumorigenesis, suggesting the observed findings might also be due to reduced amounts of recycling glutamine within the cells as they are incapable to reprocess glutamine based on their impaired autophagic capacity. Avalos and others have therefore speculated that while autophagy in normal cells and cells undergoing malignant transformation is tumor-suppressive as it abrogates the signals inducing further malignant transformation, once a threshold has been crossed, it becomes a mainly pro-tumorigenic phenomenon, as it enables the cancerous cells to endure nutrient starvation and hypoxia. However, beclin 1 and most other autophagy-related players (ATGs) as well as p62 (reviewed in [55]) have autophagy-independent functions that, in some cases, tie in with their functions in autophagy. Under conditions of inhibited autophagy execution, e.g., there is an accumulation of unutilized p62, which, in turn, influences non-autophagic pathways, such as the nuclear factor kappa-light-chain-enhancer of activated B cells (NF-κB) for tumor promotion [56]. Beclin 1, for example, has been shown to modulate p53 levels by an autophagy-independent mechanism [57]. Overall, this makes a definitive causative and isolated relationship between autophagy and the observed effects impossible to prove at the moment.

### 2.2. Oncogenic Functions of Autophagy

Malignant cells are defined by increased proliferation, which comes with an increased energy demand. This oftentimes collides with the fact that fast-growing tumors lack qualitatively sufficient blood vasculature and have therefore large areas in which a lack of nutrients, as well as hypoxia, define and shape the cellular environmental conditions.

Degenhardt and White described that autophagy was crucial for tumor cell survival under metabolically challenging conditions, especially in the frontier regions of tumor vasculature, i.e., near the tumor center, where conditions are hypoxic [42]. They used a tumor model system based on immortalized baby mouse kidney epithelial cells expressing either BAX/BAK (W2) or being deficient for BAX/BAK (D3) that they had described previously [58]. In the current study, the authors manipulated those cells to either constitutively express AKT (myr-AKT) or RAS (H-rasV12) and injected these cells into nude mice subcutaneously. However, they also showed that blocking autophagy in these apoptosis-defective cells was detrimental to the tumor cells themselves by leading to necrosis but led to effects beneficial to the tumor as a whole by promoting progression based on necrosis-triggered inflammation.

As outlined above and eloquently reviewed by White in 2012, central aspects of the mechanisms by which autophagy supports survival for cancer cells to withstand hypoxia and nutrient depletion revolve around the biological functions of mitochondria [59]. This is crystallized in the well-studied dependency of Ras-driven tumors, such as pancreatic cancer, on autophagy, which has also been reviewed by Avalos and colleagues [54]. It has been demonstrated that pancreatic ductal adenocarcinoma (PDAC) patients have a worse prognosis if there is evidence of strong autophagy in the tumor tissue [60], suggesting a biological benefit for the pancreatic tumor through autophagy. Further studies provided evidence that increased autophagy observed in those cells prevented the accumulation of reactive oxygen species (ROS) especially from damaged mitochondria, limiting associated damage of e.g., DNA [43,61]. While autophagy inhibition by RNA interference or pharmacological means in form of chloroquine (CQ) proved to inhibit PDAC tumor growth both in vitro and in vivo, the authors found that not alteration of mitophagy but indeed autophagy-based alteration of mitochondrial metabolism appeared to be the cause for the observed changes, as a failure to supply “fuel” for the tricarboxylic acid (TCA) cycle by means of autophagic recycling hampered the capacity of mitochondria to perform oxidative phosphorylation [43]. Elaborating on these findings in a review [59], White laid out that RAS activation reduces the provision of acetyl coenzyme A (acetyl-CoA) by reducing pyruvate availability through several means: reducing pyruvate dehydrogenase (PDH) activity and instead additionally favoring the conversion of pyruvate to lactate by the lactate dehydrogenase (LDH) by a process involving hypoxia-inducible factors [62,63]. Furthermore, another crucial supply route for the TCA, lipids, appears to be dependent on functioning autophagy, as suggested by data from a mouse model using K-Ras-induced lung tumors [44]. In this study, an Atg7 knock-out provided further evidence for the observation that tumorigenesis based on RAS is dependent on functioning autophagy for tumor proliferation, especially under conditions of starvation. The authors also observed an interesting link between autophagy and lipid metabolism in tumors with activating RAS mutations and concomitant deletion of p53. In those tumors, interruption of autophagy by Atg7 knock-out lead to a phenotype with a reduction in β-oxidation with the subsequent accumulation of lipids and mitochondria with impaired functionality [44]. Complementary data from a model using a knock-out of the tumor-suppressive liver kinase B1 (LKB1), which is frequently co-mutated in RAS-driven lung carcinomas in humans [64], substantiated that Atg7-based autophagy deficiency in those cancers with double-deficiency abrogated their progress in vivo, as cancer cells were unable to cope with challenging nutrient-depleted conditions [65]. Under those conditions, autophagy was a crucial lifeline ensuring ongoing metabolic activity by providing amino acids, such as glutamine, to keep the TCA cycle running. Autophagy inhibition via Atg-7 knock-out forced the cells to quickly burn through their lipid reserves by increased β-oxidation, leading to what the authors termed an “energy crisis” [65]. This ties in with the observations of other researchers providing evidence of AMPK-based positive regulation of lysosome formation in RAS-driven lung tumors as a means of tumor survival [66], an interesting finding in the context of the previously described inductive function of AMPK regarding β-oxidation in normal tissues [67]. Furthermore, recent research suggests that acute myeloid leukemia (AML) cell lines, in contrast to normal cells, depend on intact oxidative phosphorylation to convey a feedback loop using mitochondria–endoplasmic reticulum contact sites (MERCs) to induce autophagy in order to degrade lipid stores and ensure the supply of lipid metabolites for energy production, sustaining cancer cell survival [45]. Although these data were obtained in a different model system, they complement the observations of autophagy determining the rate of β-oxidation made previously in lung tumors [44] by shedding more light on the complex regulatory interplay between the metabolic function of mitochondria and autophagy regulation. Beyond this, new evidence has also emerged for autophagy-dependence as an oncogenic mechanism in colorectal carcinomas (CRC). While Devenport and colleagues corroborated the principle of autophagy supplying the needed metabolic intermediates for mitochondria, similar to the findings in the other tumor entities using metabolomic studies, they also provided evidence of PINK1-mediated mitophagy, i.e., direct mitochondrial recycling as a survival mechanism of colorectal cancer cells [46].

The well-known original Warburg effect refers to discoveries departing from Otto Warburg’s original “Warburg hypothesis” that cancer is caused by dysfunction of the mitochondria [68]. While this assumed causal relationship has not stood the test of time, the accompanying observation that cancer cells produce energy by, e.g., lactic acid fermentation despite the presence of oxygen (a situation called “aerobic glycolysis” by Warburg), has. Current understanding has expanded this observation in a fashion that cancer cells not only perform increased lactic acid fermentation despite the presence of oxygen but also despite the presence of intact mitochondria and oxidative phosphorylation. In a seminal review, Vander Heiden and colleagues coined the hypothesis that this modern Warburg effect in multicellular organisms is independent of nutrient resources but instead conditioned by a “proliferative metabolism” caused by growth signals found in quickly proliferating normal tissues and cancer cells alike to create energy and building blocks for further cellular proliferation [69].

This intricate relationship between growth signals and metabolism is also found in the concept of the “reverse Warburg effect”, in which autophagy is postulated to play a crucial role. This comparatively new and compelling concept, previously described by [70] and conceptually reviewed in detail by Pavlides and colleagues [71], suggests that tumor cells “corrupt” [70] peritumoral stromal cells for their own needs. While the knock-out of caveolin-1 has been shown to increase autophagy and mitochondrial ROS in endothelial cells [72], Mercier and Pavlides found that reduced caveolin-1 was a hallmark of cancer-associated fibroblasts (CAF) in breast cancer patients [73]. Accordingly, they used a caveolin-1 knock-out mouse model to describe that those CAFs showed increased glycolysis [70]. Then, in their review “Warburg Meets Autophagy” [71], they laid out the case that epithelial breast cancer cells employ reactive oxygen species (ROS) to trigger lasting metabolic changes in neighboring fibroblasts, in particular caveolin-1 downregulation by autophagic degradation [47], mitochondrial dysfunction via NF- κB, and hypoxia-inducible factor 1-α (Hif1-α) activation together with induction of autophagy, priming the CAFs to a catabolic state employing aerobic glycolysis [48,49]. Amino acids such as glutamine, lactate, pyruvate, and ketone bodies are then supplied to the adjacent cancer cells (reviewed in [74]). This metabolic servantry presents an important advantage for the tumor as it facilitates oxidate phosphorylation and proliferation, resulting in a correlation of poor prognosis with the loss of caveolin-1 in breast cancer [75]. Recent evidence has suggested that this co-dependency of tumor cells and CAFs is also highly relevant in other organs such as the lung [76] and the prostate [77], with the latter publication emphasizing that this metabolic reprogramming is a bidirectional relationship. A computational study also suggested that while the reverse Warburg effect proved to be advantageous for tumors, a so far not yet described glutamine utilization outside the TCA might be an important underlying mechanism in this scenario [78].

### 2.3. Autophagy-Mediated Immune Evasion

Evading the antitumoral immune response is an important survival tactic employed by various tumors. Recent evidence suggests that autophagy in its function of disassembling proteins plays a major role in these tumor immune evasion maneuvers. Yamamoto and colleagues recently described that downregulation of MHC class I molecules on pancreatic ductal adenocarcinoma (PDAC) was mediated by selective autophagy-based degradation and that autophagy inhibition unleashed a strong anti-tumor immune response (mediated by T cells) based on the reappearance of the MHC class I molecules on the cancer cells in vivo [50,51]. On the other side of the anti-tumoral immune response, within the leukocyte compartment, specific myeloid-derived suppressor cells (MDSCs) act as rogue agents in cancer by blocking a robust anti-tumoral immune response from being carried out. Alissafi and colleagues recently offered proof that autophagy in MDSCs is a crucial mechanism by which they silence anti-tumor immune activity in melanomas. In analogue fashion to the findings in epithelial cancer cells, autophagy in MDSC immune cells was central to degrading MHC class II molecules, preventing priming and activation of anti-tumoral T cells (CD4^+^). Accordingly, autophagy inhibition using chloroquine proved to disinhibit anti-tumoral T cell activity [52]. A more complex role in CD8^+^ T cell anti-tumor activity was recently described by DeVorkin et al. In this study, blocking autophagy by murine knock-out of Atg5 or other central autophagy players in CD8^+^ T cells resulted in a vastly improved anti-tumor immune activity by those cells. Underlying to this was an induction of the glucose metabolism, leading to complex changes and resulting in an effector T cell phenotype [79]. This not only illustrates the importance of autophagy in the anti-tumor immune response but again emphasizes the complicated regulatory environment interlinking autophagic processes and their regulation with metabolic and proliferative aspects of (cancer) cell function in different cell types. Focusing on tumor-associated macrophages, Cunha et al. described LC3-Associated Phagocytosis (LAP) of demised tumor cells as another vantage point of suppressing anti-tumor T cell activity by facilitating M2 macrophage polarization, resulting in an abrogated T cell response to the actual tumor [80]. This adds yet another layer of intricacy, as it demonstrates that not only autophagy as a process itself but also proteins centrally involved in autophagic pathways such as LC3 play a role in other mechanisms such as phagocytosis. This has to be kept under consideration in potentially exploiting this mechanism for the disinhibition of anti-tumoral immune activity as potential anti-tumor treatment, as possible side effects of LC3 inhibition could reach far into basic autophagic functions. Opposite to these findings, Li et al. present evidence that polyubiquitination-based p62/LC3-mediated autophagic degradation of the protein tenascin-C in triple-negative breast cancer cells leaves them vulnerable to the killing by cytotoxic T cells, showing a clear tumor-suppressive function of autophagy based on anti-tumoral immune activity. Accordingly, impairments and defects of this autophagy pathway in the wild or its abrogation by pharmacologic and genetic means led to relevant presentation of tenascin-C on the tumor cell surface, inhibiting the T cell response and yielding a clear advantage to the immune evasion of triple-negative breast cancer [81].

In summary of the above highlighted study data, autophagy can exert both oncogenic as well as tumor-suppressive functions, with its exact role apparently depending on the spatiotemporal context, cell type, cellular compartment, as well as a complicated interplay within wider metabolic and proliferative regulatory networks.

### 2.4. Autophagy and Therapy Resistance

The observations outlined above focus on the role of autophagy in the spontaneous course of cancer development and progression. However, treatment of malignant diseases by either conventional chemotherapies or new therapies, including antibodies and small molecules, brings a new and dynamic element into this framework. Autophagy, in its role as a mechanism for (cancer) cells to cope with threatening stressors, has been described as an important mechanism of therapy resistance in cancer treatment [82]. As also reviewed by Chavez-Dominguez et al., evidence has been provided that tumor cell resistance against cisplatin is, at least in part, mediated by increased autophagy in ovarian cancer cell lines [83]. Similar evidence exists for cisplatin, doxorubicin, and methotrexate for overcoming chemoresistance by inhibiting autophagy in osteosarcoma [84]. Interestingly, as reviewed by Pérez-Hernández and colleagues [85], other studies also suggest that the interplay between e.g., cisplatin and autophagy, represents a continuum and that adding autophagy inhibitors such as CQ also enhances therapeutic efficacy, even in cells not per se classified as resistant to certain chemotherapeutic drugs in vitro and in vivo. This has been demonstrated in a murine model of adrenocortical carcinoma [86], for cell lines of colon cancer and 5-fluorouracil [87], and for temozolomide-induced cytotoxicity in glioma cells [88]. Beyond that, similar observations have been made for antibody-based therapies such as in trastuzumab-resistant breast cancer cells, in which autophagy inhibition using CQ led to an almost complete abrogation of tumor growth in a previously completely trastuzumab resistant tumor [89]. Along the same lines, inhibiting autophagy that occurs in glioblastomas in response to the confrontation with bevacizumab treatment employing HCQ led to increased cell death [90]. Autophagy inhibition using CQ was also able to effectively counter bevacizumab-induced induction of autophagy in colorectal cancer cells, reducing tumor growth in an in vivo murine tumor model [91].

## 3. Autophagy in Cancer Treatment—Current Status and Perspectives

### Clinical Trials

Despite the complexities of autophagy in the development and progression of malignant tumors, inhibition of autophagy in the treatment of already established and oftentimes systemically metastasized tumors, in particular of the pancreas, lung, breast, and colon, is the predominant focus of recent and current clinical trials. This is also based on the fact that chloroquine (CQ) and its derivate hydroxychloroquine (HCQ) are established inhibitors of autophagy, widely used in in vitro and in vivo experiments, but are also approved for human use in diseases such as malaria [92] and autoimmune diseases such as systemic lupus erythematosus [93]. A recent detailed analysis of their molecular mechanism has suggested that its main point of action is the fusion step between the autophagosome and the lysosome and that both CQ and HCQ have additional effects not directly linked to the execution of autophagy causing, as the authors phrased it, “severe disorganization” in other organelles such as the Golgi apparatus [94]. Additionally, the recent experiences of the medical community with CQ and HCQ in the treatment of COVID-19, in which it proved counterproductive and even dangerous, as evidenced by a recent meta-analysis detailing increased mortality rates in hospitalized patients treated with HCQ for COVID-19 [95], suggest caution in transferring effects from in vitro to in vivo and from one disease entity to another. However, a recent meta-analysis based on seven studies using CQ or HCQ in combination cancer treatments further substantiated previous results from clinical trials, demonstrating significant improvements in key outcome parameters, including overall response rate and 1-year overall survival [96].

In accordance with the promising preclinical data regarding autophagy inhibition in pancreatic ductal adenocarcinoma (PDAC), there are several published phase II and one phase I/II study using hydroxychloroquine [97,98,99,100]. An early phase II study from 2014 used HCQ monotherapy in patients suffering from metastatic pancreatic cancer that had previously been treated by other means with a primary endpoint of two months of progression-free survival [99]. As a result, there were variably reduced levels of autophagy in different patients, as evidenced by a surrogate parameter (LC3-II in circulating PMNs), but there was no significant improvement in the primary endpoint. However, this study used HCQ monotherapy in patients with advanced and pretreated disease. Still, another study combining HCQ with Gemcitabine and nab-Paclitaxel in patients with either advanced or metastatic pancreatic cancer also failed to demonstrate prolonged overall survival at 12 months. Importantly, however, patients with an HCQ including therapy regimen displayed a vastly and significantly better response rate (38.2% vs. 21.1%) [97]. This led the authors to speculate that HCQ might be useful in downsizing pancreatic cancer before surgical therapy. Indeed, one study evaluating the response on preoperative pancreatic adenocarcinoma using HCQ in combination with gemcitabine found that a more than 51% reduction in autophagy, as determined by the same surrogate parameter described above (LC3-II in circulating PMNs), was correlated with a significantly (*p* < 0.05) longer disease-free survival and overall survival in HCQ-treated patients, with the p53 status not apparently relevant to the outcome [100]. Beyond that, a more recent study from the same group evaluated neoadjuvant regimens of gemcitabine and nab-Paclitaxel with or without additional HCQ in patients with pancreatic cancer [98]. Complementing the previous study that focused on survival, this study focused on the histopathological tumor response, as previously described by Evans et al. [101], as the primary endpoint among others. Analysis revealed that removed tumors that had been additionally confronted with HCQ had a better Evans grading, more immune cells within the tumor, as well as accumulated p62 as a surrogate parameter of reduced autophagy execution. Unfortunately, neither relapse-free nor overall survival was significantly improved in this study. The authors argue that this was probably due to differences in adjuvant treatment, which deviated from the current standard FOLFIRINOX in all but three patients. Follow-up studies will definitely be necessary to evaluate whether this plausible speculation is indeed true and addition of HCQ early and constantly during treatment might improve neoadjuvant tumor response, resectability, and overall survival. Importantly, the finding of an increased anti-tumoral immune response in this phase II study [98] is in line with the observations of autophagy-based immune evasion of pancreatic cancer by a reduction in MHC class I molecules in a preclinical murine model [50]. In this study, autophagy inhibition with chloroquine in mice in vivo led to a reappearance of MHC class I molecules on the tumor cell surface and an augmented anti-tumoral immune response with consecutively reduced tumor burden. Fascinatingly, inhibition of autophagy also made these murine tumors susceptible to dual immune checkpoint inhibition with anti-PD1 and anti-CTLA4, which was ineffective against non-chloroquine-treated tumors. Together, these findings suggest new and exciting possible avenues, such as combining immune checkpoint therapies with autophagy inhibition in clinical trials for the treatment of pancreatic cancer.

Clinical phase II studies focusing on other disease entities are heterogenous with regard to the tumor entities studied. First initial encouraging results are available from some trials, while others show disappointing results. Patients with chronic myeloid leukemia in major cytogenetic remission but still present disease activity as evidenced by qPCR were treated using a combination regimen of HCQ and imatinib or imatinib alone in the CHOICES trial [102]. While the primary endpoint, a reduction greater than or equal to a 0.5 log decrease in disease burden 12 months in, was not reached, the secondary endpoint for this analysis at 24 months was higher in the HCQ-containing group with a strong trend although not statistically significant. Although the authors speculate that insufficient HCQ plasma concentrations might have been responsible for failing the primary endpoint, these data are still highly interesting against the backdrop of in vitro data from (acute) leukemia cells, which suggest an important metabolic disadvantage towards leukemic cells under conditions of autophagy inhibition [45]. Furthermore, data from metastatic NSCLC patients combining HCQ with carboplatin, paclitaxel, and facultatively bevacizumab suggest better progression-free survival for patients with the autophagy inhibitor [103]. While these data complement preclinical results using Ras-driven lung carcinomas previously described in this review [65,66,67], more rigorous study designs will be needed to substantiate any real-world benefit for patients. Similarly, previously treated renal cell carcinoma patients were treated with everolimus and HCQ, showing a slightly longer survival [104]. This study vividly illustrates the complexities of combining autophagy inhibitors with established treatments: The authors in the study focusing on renal cell carcinoma failed to detect evidence of accumulation of autophagic vesicles in the common surrogate measurement in PBMCs despite this being the case in a study using comparable concentrations of HCQ [105]. One might speculate that this [104] was due to the combination with everolimus, which has been recently demonstrated to act as an autophagy inducer in human breast cancer [106] and other diseases [107,108]. However, the study showing evidence of autophagy inhibition was also using a combined approach with temsirolimus [105], which has been shown to be an autophagy inducer in vivo as well [109,110,111], suggesting other potential and yet to be elucidated pathway interactions as possible reasons. Other studies have shown no benefit and oftentimes no measurable alteration in the autophagy parameters (Table 2).

However, even with limited data suggesting a tangible survival benefit, those and similar trials are important to test the real-world biological consequences of established and new autophagy modulating concepts. A current study listed on ClinicialTrials.gov focusses, in form of a follow-up study, on advanced and metastatic pancreatic cancer in combination with gemcitabine and paclitaxel, including metabolic profiling (NCT01506973), which is an important new read-out perspective as the interlinks between autophagy and metabolism especially in proliferating (tumor) cells are manifold, as outlined above. Studies targeting breast cancer (NCT04841148, NCT03774472) in combining HCQ with other drugs, including immune checkpoint inhibitors, would also represent an interesting setting for metabolomic profiling given the initial data suggesting widespread metabolic alterations in CAFs based on autophagy [47,48,49,71,74,75]. Along the same lines, studies on prostate cancer and other solid tumors (NCT02339168, NCT05036226) use, among others, HCQ and metformin. Metformin is an interesting choice not only for the reason that it is, similarly to HCQ, an already approved drug, but because it has recently been described to prevent the allocation of glutamine by reducing the activity of the enzyme glutaminase and to block ammonia-induced autophagy in tumors cells [112]. Based on already published data supporting the hypothesis of the reverse Warburg effect in prostate cancer based on metabolic reprogramming of CAFs mediated by autophagy [77], this makes a promising approach and might help to explain the findings of a recent meta-analysis showing metformin improving key outcomes including overall survival and progression-free survival in patients with prostate cancer [113]. Other studies focus on different RAS-mutated malignancies, combining HCQ with different MEK inhibitors, such as trametinib (NCT03979651; NCT04566133), or with cobimetinib plus the immune checkpoint inhibitor atezolizumab (NCT04214418), as well as an ERK1/2 inhibitor in pancreatic cancer (NCT04386057). Another study is testing BRAF inhibition and MEK inhibition in combination with HCQ, adding to the preclinical data of Strohecker et. al. showing a potential weak point of BrafV600E-mutated lung tumors by being at least to some extent metabolically dependent on autophagy [41]. Further studies are listed in Table 3.

## 4. Conclusions and Outlook on Future Directions

Autophagy in cancer treatment is and remains a complex yet promising field of medical science with great potential. However, while clinical trials so far have offered some promising initial results using autophagy inhibition, e.g., in pancreatic cancer, other studies reported mixed or disappointing results. This might be due to general problems: While almost all trials included a (semi-)quantitative assessment of autophagy, oftentimes based on surrogate parameters such as measurement in different cell populations, no universal gold standard for autophagy analysis exists. Instead, there is a complex multifaceted analysis scheme, complicating the definition and proof of efficient autophagy inhibition [37]. Additionally, insufficient autophagy inhibition observed in some studies might very well be due to complex compensatory interplays with other regulatory networks. However, the heterogeneity of tumor entities and mutations encountered in these heterogeneous study cohorts also complicates the evaluation and understanding of causative relationships as it might be a mixture of very different reasons in different patients. Noteworthy is also that a relevant number of these clinical trials recruit from a patient collective with oftentimes advanced and systemically metastasized oncologic diseases after pretreatment. One reason for this is that, as reviewed recently by Levy and colleagues [123], autophagy appears to be a common mechanism of drug treatment resistance for both conventional chemotherapy and, e.g., tyrosine kinase inhibitors [124,125,126]. Accordingly, there is evidence strongly suggesting autophagy inhibition as a potential path to overcome several drug treatment resistances, such as demonstrated in several BRAF-mutated tumors [127,128,129]. However, using autophagy inhibition-based combination therapies in earlier stages of cancer treatment, as performed by Zeh et al. [98] with HCQ in a neoadjuvant setting in pancreatic cancer patients, might be equally promising. The results from this study suggest that strategically blocking autophagy in cancers that rely on the oncogenic function of autophagy might act as a (transient) booster to conventional neoadjuvant treatment, improving the preoperative oncologic situation despite the (potential) incapability of systemic anti-autophagic combination therapy to control the disease on its own. Overall, autophagy inhibition appears most promising as a combination therapy, sensitizing tumor cells towards other therapies such as immune checkpoint blockade [50]. Initial phase II studies on renal cell cancer [104] and metastatic NSCLC [103] have also suggested longer than expected survival. This opens up the question, as in oncology in general, whether there are subgroups of patients that might show a clearer benefit than the general collective. However, due to the complex networks regulating autophagy, transferability from the preclinical to the clinical context oftentimes resulted in contrary or conflicting results, stressing the importance of explorative clinical trials in the future.

Another fascinating aspect of autophagy in cancer treatment is the integration of autophagy as a facilitator of tumor metabolism as in the “reverse Warburg effect” described above. While this and similar mechanisms represent a critical advantage for the tumor, it also creates a potentially exploitable critical vulnerability. Gremke et al. studied lung cancer cells in vitro and in xenograft tumor experiments in vivo in mice. They observed that cisplatin resistance was mediated by an increased mTOR activity, which also led to suppression of autophagy via the mTORC1-ULK1 pathway. This, however, in turn made tumor cells more susceptible to an attack on their metabolism using an inhibitor of glycolysis (2-deoxy-D-glucose2, 2DG), an inducer of pyruvate dehydrogenase (dichloroacetate, DCA), and metformin, which also acts as an inhibitor of oxidative phosphorylation via alteration of complex I [130], with all substances leading to cell death [131]. In this particular context, it appears plausible that the inhibition of oxidative phosphorylation by metformin is complementary to its assumed function in prostate cancer [112], as autophagy was already inhibited in this setting. In conclusion, there is a rapidly expanding and evolving understanding of the molecular mechanisms of autophagy in cancer and cancer therapy based on sophisticated preclinical and clinical studies, including -omics approaches, putting an increasing and necessary emphasis on the interconnectedness of the autophagy network and its regulation into wider cellular networks, such as cell metabolism and proliferation. Despite mixed results in some clinical trials, there is so far convincing evidence to support a beneficial role for autophagy modulation/inhibition in certain cancers such as pancreatic adenocarcinoma [98,100]. However, to support further clinical studies and evaluate the whole potential of this approach, there is a need for consensus and standardization regarding assessment of autophagic flux and its modulation in these studies. Beyond that, new and more specific autophagy inhibitors and/or inducers will potentially allow a more circumscriptive intervention, reducing the risk of side effects. A recent preclinical study described palmitoyl-protein thioesterase 1 (PPT1), a lysosomal enzyme whose expression is correlated with a poor prognosis in cancer patients, as the point of attack for the autophagy modulating and antineoplastic activity of CQ and its derivative DC661 [132]. Interestingly, PPT1 inhibition by HCQ or DC661 also led to an increased effect of anti-PD-1 therapy in melanoma in vitro and in a murine in vivo model [133], echoing findings recently made in pancreatic cancer [50]. After initial results suggesting antineoplastic activity against cholangiocarcinoma in vitro and in vivo in a murine model [134], the novel PPT1 inhibitor GNS561 is now evaluated in a clinical trial in combination with monalizumab, a checkpoint inhibitor, and avdoralimab, a CD88 blocker, in cancer patients (NCT04333914; Table 2), holding promise for others to follow.

## Figures and Tables

**Table 1:** Selected tumor-suppressive and oncogenic functions of autophagy in cancer and the corresponding regulators.

Experimental Context	Mode of Action	Involved Components/ Regulators	Outcome	Conclusion	Reference
Sporadic breast, ovarian, and prostate cancer	Beclin-1 facilitates autophagy	Beclin 1 (beclin1+/−) heterozygous knock-out leads to reduction of autophagy (mice)	Disruption of beclin 1 increased frequency of spontaneous malignancies acceleration of hepatitis B virus-induced premalignant lesions	Beclin1 mediated autophagy acts tumor-suppressive	[39]
Liver neoplasms	Autophagy is required for detoxification of oxidative stress and prevention of associated damage	Mosaic deletion of Atg5 OR liver-specific Atg7−/− (mice)	Lack of Atg7 in hepatocytes causes: autophagy-deficiency swelling of mitochondria buildup of p62 oxidative stress neoplastic lesions Resulting neoplasms were benign, not malignant	Autophagy acts tumor-suppressive	[40]
BrafV600E-induced lung cancer model	Autophagy causes prevention of ROS accumulation (from damaged mitochondria); autophagic recycling supports mitochondrial tricarboxylic acid (TCA) cycle/oxidative phosphorylation	Atg7 knock-out (mice)	Atg7 knock-out: induction of oxidative stress promotion of tumor formation/proliferation contrasting long term effect: reduced tumor burden, decreased proliferative capacity	Autophagy in BrafV600E tumors initially acts oncogenic and subsequently tumor-suppressive	[41]
Immortalized baby mouse kidney epithelial cells [BAX/BAK (W2) or deficient BAX/BAK (D3)]	Autophagy in apoptosis-defective cells prevents necrosis	Constitutive expression of AKT (myr-AKT) OR RAS (H-rasV12) (murine model)	Blocking autophagy causes necrosis and inflammation	Autophagy acts oncogenic regarding tumor cell survival but its inhibition leads to overall progression based on necrosis-triggered inflammation	[42]
Pancreatic ductal adenocarcinoma (PDAC)	Autophagic recycling is required to supply the tricarboxylic acid (TCA) cycle and oxidative phosphorylation	RNA interference of Atg5 OR chloroquine (CQ) (cell lines, murine model)	Blocking autophagy inhibits PDAC tumor growth both in vitro and in vivo	Autophagy acts oncogenic in RAS-dependent PDAC	[43]
K-Ras induced lung tumors OR liver kinase B1 (LKB1) knock-out (mice)	Autophagy provides e.g., glutamine for the TCA cycle	Atg7 knock-out (murine model)	Blocking autophagy leads to depleted energy metabolism with non-sustainable increased β-oxidation	Autophagy acts oncogenic in K-Ras-dependent lung tumors and liver kinase B1 mutated tumors	[44]
Acute myeloid leukemia (AML)	Feedback loop: oxidative phosphorylation induction of autophagy degradation/supply of lipid stores oxidative phosphorylation	Mitochondria-endoplasmic reticulum contact sites (MERCs) modulate autophagy (cell lines)	Mitochondria-endoplasmic reticulum contact sites (MERCs) modulate autoph-agy (cell lines)	Autophagy acts oncogenic	[45]
Colorectal cancer	Autophagy supplies metabolic intermediates for mitochondria. PINK1-mediated mitophagy causes mitochondrial recycling	Atg5 knock-out; RNA interference PINK1 (cell lines and murine model)	Blocking autophagy/mitophagy reduces tumor growth	Autophagy and mitophagy act oncogenic	[46]
Breast cancer	Caveolin-1 downregulation by ROS-induced autophagy in cancer associated fibroblasts (CAFs)	Caveolin-1 knock-out (mice)	Autophagy in CAFs leads to catabolism and supplies e.g., glutamine to adjacent cancer cells	Autophagy acts oncogenic	[47,48,49]
Pancreatic ductal adenocarcinoma (PDAC)	Autophagy degrades MHC class I molecules	Atg4B OR Atg7 RNA interference	Blocking autophagy leads to MHC class I molecule reappearance leading to increased immune detection of the tumor	Autophagy acts oncogenic	[50,51]
Melanoma	Autophagy degrades MHC class II molecules leading to myeloid-derived suppressor cells (MDSCs) blocking anti-cancer immune response	Atg5 knock-out (mice)	Blocking autophagy leads to MHC class II molecule reappearance and subsequent priming of anti-cancer leukocytes	Autophagy acts oncogenic	[52]

**Table 2:** Selected clinical studies with published results.

Autophagy-Inhibitor	Combination with	Tumor Entity	Outcome	Clinical Phase	Number of Patients	Reference
HCQ	Gemcitabine, nab-Paclitaxel	Pancreatic cancer (metastatic or advanced)	Primary endpoint: 12-month overall survival not improved. Improvement in overall response rate.	II	112	[97]
HCQ	Gemcitabine, nab-Paclitaxel	Pancreatic cancer (potentially resectable)	Primary endpoint: histological response at resection improved. HCQ led to increased autophagy-inhibition and immune activity in the tumor.	II	64	[98]
HCQ	None	Pancreatic cancer (previously treated and metastatic)	Inconsistent autophagy inhibition. No survival benefits.	II	20	[99]
HCQ	Gemcitabine	Pancreatic cancer (adenocarcinoma, preoperative)	Patients with >51% reduction of autophagy (surrogate: LC3-II in circulating PMNs) had significant (*p* < 0.05) improvement in disease-free survival (15.03 vs. 6.9 months) and median overall survival (34.83 vs. 10.83 months).	I/II	35	[100]
HCQ	Imatinib	Chronic myeloid leukemia (major cytogenetic response with residual disease)	12 months: ’Success’ rate not improved. Major Molecular Remission (MMR): 80% (Imatinib) compared to 92% (Imatinib/HCQ) (n.s.). 24 months: ’Success’ rate increased 20.8% for Imatinib/HCQ vs. Imatinib (n.s.).	II	62	[102]
CQ	None	Breast cancer (preoperative)	No effect on cancer cell proliferation (n.s.).	II	70	[114]
HCQ	Everolimus	Clear-cell renal cell carcinoma (previously treated)	Longer stable disease in some patients, inconsistent autophagy inhibition.	I/II	38	[104]
CQ	Whole-brain irradiation	Brain metastases	Overall response rate (ORR): CQ 54% vs. Control 55% (n.s.). Progression-free survival: CQ 83.9% (95% CI 69.4–98.4) control 55.1% (95% CI 33.6–77.6). CQ significantly improves PFS: RR 0.31 (95% CI [0.1–0.9]). No difference in response rate or overall survival.	II	73	[115]
HCQ	Carboplatin, Paclitaxel, Bevacizumab (if criteria met)	NSCLC (metastatic and untreated)	Progression-free survival longer than expected.	Ib/II	40	[103]
HCQ	Radiation therapy; concurrent, adjuvant Temozolomide	Glioblastoma multiforme (newly diagnosed)	No significant improvement in overall survival. Significant but inconsistent autophagy inhibition.	I/II	92	[116]
HCQ	Temsirolimus	Advanced solid tumors and melanoma	No significant improvements	I	40	[105]
HCQ	Sirolimus	Lymphangioleiomyomatosis	No improvement of lung function	I	14	[117]
HCQ	Bortezomib	Multiple Myeloma (relapsed, refractory)	Very good partial responses (14%), minor response (14%), temporary stable disease (45%)	I	25	[118]
HCQ	MK-2206 (AKT inhibitor)	Advanced solid tumors	Stable disease 15%. No significant antineoplastic activity.	I	35	[119]
HCQ	Temozolomide	Advanced solid tumors and melanoma	Metastatic melanoma: Partial response 14%, stable disease 27%. Subgroup analysis refractory BRAF wild-type melanoma: 2/6 patients almost complete response, prolonged stable disease. Significant inhibition of autophagy.	I	40	[120]
HCQ	Erlotinib	NSCLC (advanced, prior clinical response to EGFR-TKI)	No relevant toxicities.	I	27	[121]
HCQ	Sirolimus, Vorinostat	Advanced Cancers	Partial response: Refractory Hodgkin lymphoma; perivascular epithelioid tumor. Stable disease: Hepatocellular carcinoma, fibromyxoid sarcoma.	I	70	[122]

**Table 3:** Selected ongoing interventional trials listed on ClinicalTrials.gov (* actual and ** estimated enrollment).

NCT Number	Tumor Entity	Autophagy Modulator	Combination with…	Clinical Phase	Enrollment	Registration
NCT03037437	Hepatocellular cancer	HCQ	Sorafenib	II	68 **	https://ClinicalTrials.gov/show/NCT03037437, accessed on 1 October 2021
NCT04214418	Gastrointestinal cancer	HCQ	Cobimetinib, Atezolizumab	I/II	175 **	https://ClinicalTrials.gov/show/NCT04214418, accessed on 1 October 2021
NCT04386057	Pancreatic cancer (advanced)	HCQ	LY3214996	II	52 **	https://ClinicalTrials.gov/show/NCT04386057, accessed on 1 October 2021
NCT05036226	Prostate Cancer (recurrent), solid tumors	HCQ	Metformin, Sirolimus, Nelfinavir, Dasatinib	I/II	76 **	https://ClinicalTrials.gov/show/NCT05036226, accessed on 1 October 2021
NCT02339168	Prostate cancer	Metformin	Enzalutamide	I	24 *	https://ClinicalTrials.gov/show/NCT02339168, accessed on 1 October 2021
NCT01506973	Adenocarcinoma (advanced, metastatic)	HCQ	Gemcitabine, Abraxane	I/II	119 *	https://ClinicalTrials.gov/show/NCT01506973, accessed on 1 October 2021
NCT04566133	Cholangiocarcinoma	HCQ	Trametinib	II	30 **	https://ClinicalTrials.gov/show/NCT04566133, accessed on 1 October 2021
NCT02042989	Advanced cancers		MLN9708, Vorinostat	I	68 *	https://ClinicalTrials.gov/show/NCT02042989, accessed on 1 October 2021
NCT01023737	Malignant solid tumors	HCQ	Vorinostat	I	72 *	https://ClinicalTrials.gov/show/NCT01023737, accessed on 1 October 2021
NCT04333914	Hematological or solid tumor (advanced, metastatic)	Autophagy inhibitor (GNS651)	Standard of care, Avdoralimab, Monalizumab	II	219 **	https://ClinicalTrials.gov/show/NCT04333914, accessed on 1 October 2021
NCT03774472	Breast Cancer	HCQ	Letrozole, Palbociclib	I/II	54 **	https://ClinicalTrials.gov/show/NCT03774472, accessed on 1 October 2021
NCT01480154	Malignant solid neoplasms (advanced), cutaneous melanoma, prostate cancer, renal cell cancer	HCQ	Akt Inhibitor MK2206	I	62 *	https://ClinicalTrials.gov/show/NCT01480154, accessed on 1 October 2021
NCT04527549	Melanoma (advanced)	HCQ	Placebo, Trametinib, Dabrafenib	II	84 **	https://ClinicalTrials.gov/show/NCT04527549, accessed on 1 October 2021
NCT04841148	Breast cancer	HCQ	Avelumab, Palbociclib	II	96 **	https://ClinicalTrials.gov/show/NCT04841148, accessed on 1 October 2021
NCT03979651	Melanoma (metastatic NRAS)	HCQ	Trametinib	N/A	29 **	https://ClinicalTrials.gov/show/NCT03979651, accessed on 1 October 2021
NCT03598595	Osteosarcoma (recurrent, refractory)	HCQ	Docetaxel, Gemcitabine	I/II	31 **	https://ClinicalTrials.gov/show/NCT03598595, accessed on 1 October 2021
